# “Playing helps you feel at home”: recovery and reintegration of child victims of political and social violence

**DOI:** 10.3389/fpsyg.2025.1607643

**Published:** 2025-09-18

**Authors:** Aleksandra Głos, Lina Martín Corredor, Ivonne Vargas Celis, Verónica Monreal, Antonia Valenzuela Sarrazin

**Affiliations:** ^1^Faculty of Medicine, Pontifical Catholic University of Chile, Santiago, Chile; ^2^Faculty of Law, Pontifical Catholic University of Chile, Santiago, Chile; ^3^Department of Special Education, Early Childhood, and Culturally & Linguistically Diverse Education, School of Education, Metropolitan State University of Denver, Denver, CO, United States; ^4^School of Nursing, Faculty of Medicine, Pontifical Catholic University of Chile, Santiago, Chile; ^5^Faculty of Psychology, The Pontifical Catholic University of Chile, Santiago, Chile; ^6^Mondes Américains, University of Paris Nanterre, Paris, France

**Keywords:** play, right to play, political and social conflict, child migrants and refugees, peace, trauma, resilience, participation

## Abstract

**Introduction:**

Play is a fundamental right of every child and one of their most important needs. However, despite the countless benefits of play for children's wellbeing and development, it is often overlooked in public policies, and especially so in the situation of political conflict and social unrest.

**Methods:**

Drawing from the children's rights framework, this qualitative study explores the role of play for the psychological recovery and social reintegration of children affected by political and social violence. It does so within the wider framework of peace studies and includes the exploration of children's conceptions of peace and the question of how play can contribute to promoting a culture of peace in violence-stricken societies. Through play-based focus groups with child migrants and refugees in Chile, we explored their own views on the therapeutic and peace-building potential of play as a response to self-reported problems.

**Results:**

The children in our study told a dual story: a story of trauma and violence, and another one about hope, friendship, and resilience, all of which were greatly facilitated by play.

**Conclusion:**

For that reason, as representatives of children's vulnerable voices, we advocate for the greater and more systematic provision of children's right to play in conflict zones and its inclusion in peace-building programs.

## Introduction

Play is one of the most important needs, pleasures, and goods of childhood. It is also a right of the child: both a natural right (Finnis, 2011) and a basic human capacity (Nussbaum, 2013). According to Article 31 of the United Nations Convention on the Rights of the Child (UNCRC), governments must ensure and promote this right regardless of children's nationality, race, religion or abilities (Art. 2). Recognizing the value of play as a specific good and right of childhood marks a transition to the understanding of childhood as a space of “being” and not only “becoming” future adults (Arneil, 2002). The Committee of the Rights of the Child distinguishes the intrinsic from the instrumental value of play (CRC, 2013). The intrinsic value refers to the importance of play for the pleasure and dignity of childhood. In an adult-governed world, play is one of the few spaces where children can exercise their agency, liberty, and creativity in inventing their own scenarios, worlds, and characters. As it is well-evidenced in literature (Pellegrini, 2009; Milteer et al., 2011; Gray, 2013; Yogman et al., 2018), play also has an instrumental value as “an essential component of the physical, social, cognitive, emotional, and spiritual development” of children (CRC, 2013, p. 14).

However, despite the countless benefits of play for children's wellbeing and development, play is often overlooked in public policy, academic research, and individual interventions. It is traditionally described as a forgotten (Hodgkin and Newell, 2007; Fronczek, 2009) and neglected (Collins and Wright, 2019) right that has only recently begun to attract more academic attention (Lott, 2023). Political conflict and social unrest constitute a situation where children's right to play is easily ignored and overrun by other, apparently more important and urgent needs and rights (cf. Collins and Wright, 2019). Although many international organizations and NGOs do provide numerous play-based initiatives in conflict zones and refugee camps, these initiatives are rarely subject to systematic research and evaluation (Ager et al., 2013). Moreover, little is known (cf. Payà and Bantulà, 2021) about how hosting countries fulfill their obligations under UNCRC (Art. 2 and Art. 22 in relation to Art. 31) to protect migrant and refugee children's right to play. It is also unclear whether and how these initiatives attempt to identify and respond to the specific challenges faced by this vulnerable group of children.

Drawing from this human rights framework and existing research on the topic, this article aims to explore the role of play for children's physical and psychological recovery, as well as social reintegration (cf. CRC, 2013, p. 13). It does so within the wider framework of peace studies, involving an exploration of children's conceptions of peace and the question of how play can contribute to promoting culture of peace in violence-stricken societies.

## Play and peace in a human rights framework

Peace and the protection of human rights are deeply interconnected and mutually reinforcing. Although the right to peace has not yet been proclaimed, the UN Declaration on the Right to Peace (United Nations General Assembly, 2016) affirms that “enjoyment of peace” is a necessary condition for the full realization of all human rights. As stated in Article 1: “Everyone has the right to enjoy peace such that all human rights are promoted and protected, and development is fully realized.” At the same time, respecting human rights is essential for building and sustaining peace. Since human rights violations often serve as both a cause and a consequence of violent conflicts, the protection and promotion of rights can function both as a preventive measure and as a means of transforming conflict-affected societies. The UNICEF (2023) Peacebuilding Framework emphasized the central role of children's rights in fostering a culture of peace.

Similarly, the realization of children's right to play is not only protected by peace—it also contributes to peace. This contribution is affirmed by the Committee of the Rights of the Child in its General Comment on Article 31. In its paragraph 39, the Committee has emphasized the vital role of play for the “physical and psychological recovery and social reintegration of a child victim of any form of neglect, exploitation, or abuse; torture or any other form of cruel, inhuman or degrading treatment or punishment; or armed conflicts” (CRC, 2013, p. 39). While highlighting the beneficial potential of play for children's recovery and reintegration, the Committee drew particular attention to its therapeutic effects. As the Committee stated, “Opportunities to realize the rights under Article 31 can provide a valuable means through which children can externalize traumatic or difficult life experiences in order to make sense of their past and better cope with their future.” Importantly, the Committee has underscored the participatory nature of play and the agency of children in the process of trauma-recovery in this context, calling play a “natural, self-guided, self-healing process”.

The peacebuilding potential of play is consistently operationalized through multiple UNICEF Humanitarian Action Reports and Peacebuilding Frameworks. One of UNICEF's key strategies for promoting play-based trauma healing is the establishment of Child-Friendly Spaces (CFS) in post-conflict settings. These spaces are designed to provide children with a safe and structured environment in which they can express themselves, enjoy the company of peers, and begin their healing process. As UNICEF has repeatedly affirmed, play has a crucial role in creating a sense of normalcy,” and “minimizing the effects of a traumatic experience, like witnessing violence, losing loved ones or seeing one's home destroyed by shelling (UNICEF USA, 2025).” Beyond individual recovery, play also functions as a powerful tool for social reintegration and inclusion. Even if this function of play has yet to be theoretically explored and practically applied, there is evidence that it helps conflict-affected children to develop friendships, and promotes “various dimensions of social cohesion,” such as “trust, tolerance of others, and the ability to resolve disputes constructively” (UNICEF, 2016, p. 21).

In the context of Latin America, children and adolescents have been recognized as crucial agents in the achievement and sustenance of peace and as catalysts of positive social change (UNICEF Colombia, 2024). Particularly in the armed Colombian conflict, UNICEF called for a change of the traditional perspective in treating children and adolescents as passive subjects whose judgments and opinions can be dismissed. Instead, UNICEF points out that children and adolescents have natural characteristics such as creativity, energy, and idealism that position them well to contribute to the management of differences and conflict resolution (UNICEF Colombia, 2019). However, to make good use of their potential, children and youth's participation needs to be included in all strategic plans aimed at peacebuilding (Aldeas Infantiles SOS, 2021). For instance, in the Peace Agreement framework, this imperative was endorsed by a coalition of national and international organizations, including the Colombian Presidential Advisory Office for Human Rights and the International Organization for Migration, which developed a project called, “*My Future is Today: Creating Peaceful Environments for Children, Focused on Preventing Recruitment*.” In this project, 7,200 children and adolescents participated in play-based activities and art projects aiming at promoting conflict resolution skills, raising awareness about human rights, and contributing to local peace-making initiatives. Similarly, the Colombian Commission for the Clarification of Truth, Coexistence, and Non-Repetition also emphasized how indispensable it is to integrate children's and youth's perspectives in the construction of peace by carrying out the “*The National Consultation of Children and Young People for the Truth*” to foster a participation space led by children and young people to learn about their perspectives on the value of learning the truth with respect to the armed conflict in Colombia. This Latin American orientation of positioning children and youth as peace agents, combined with the use of play as a pedagogical tool of participation and reflection, is certainly a framework worthy of being replicated and expanded.

In line with the emerging UN framework on peace and play and inspiring Latin American practices of positioning children as agents of peace, we hope that our participatory research will contribute to strengthening both scientific evidence and human rights-based programs that—through play—promote children's active role in peacebuilding in all regions of the world affected by war and conflict.

## Play and peace in academic literature

Similar to the UN framework, the role of play in promoting peace remains an emerging topic within academic literature. Nevertheless, existing research increasingly supports play's contribution to the mental recovery and social reintegration of children victims of war and conflict.

### Play and inner peace

The strongest evidence for the impact of play relates to trauma recovery and mental wellbeing, which can be described in terms of play's contribution to children's inner peace. It can be considered symbolic that the roots of play therapy can be traced back to the war-related context. In the aftermath of World War II, pioneering psychologists Anna Freud and Dorothy Burlingham observed that while most children struggled to articulate their traumatic experiences, they recurrently “played it out” (Freud and Burlingham, 1943; Feldman, 2019). One such war-themed example was the “air raid” game, in which a child would shout “*Alert! Danger!”*, while others scatter and hide—as if under bombardment. While such playful reenactments may appear unsettling, they served a vital psychological function: they allowed children to externalize their trauma and process it symbolically in the company of peers. These early observations led to recognizing play as a powerful therapeutic and diagnostic tool, especially for children unable or unwilling to express their distress through verbal language. Contemporary clinical studies reinforce this therapeutic potential of play. For instance, (Cohen and Gadassi 2018) observed that, when encouraged to re-enact traumatic events through play, children can often reframe their meaning and emotional burden over time, transforming fear into mastery and helplessness into control.

Further research shows that play serves as a useful therapeutic tool for war-affected children, reducing their traumas, helping overcome behavioral and psychological challenges, in addition to improving emotional regulation and literacy (Moody-Pugh et al., 2025; Parker et al., 2021). (Schottelkorb et al. 2012) examined the effects of child-centered play therapy in refugee elementary children who experienced violent combat in their home country and were diagnosed with Post-Traumatic Stress Disorder. The researchers found that the play therapy used produced a “significant reduction in PTSD symptom severity” (p. 68), which in turn contributed to improving children's cognitive development and academic performance. In their exploratory study, (De Freitas Girardi et al. 2020) designed and implemented workshops to activate the “playful energy” of young asylum seekers in Canada. They found that symbolic play allowed participants to progressively transition from states of distress such as tantrums and volatile behavior to emotional regulation with calmed behaviors. Symbolic play also provides a safe space for children to express past and present experiences. Other studies have found evidence of similar benefits of play in promoting inner peace in traumatized children, by reducing symptoms of anxiety and depression, improving attention, cultivating appropriate emotional expressions, and reactivating positive memories (Jones, 2015; Kwon and Lee, 2018).

### Play and social peace

The role of play in fostering social peace remains underexplored in academic literature. However, some studies have evidenced the positive effects of play during and after traumatic events such as war, armed conflict, family separation, and forced displacement (e.g., Feldman, 2019; Gura and Roma, 2024). For instance, when playing, children demonstrated compassion by “consoling victims of war” (Feldman, 2019, p. 296), “protecting loved ones,” and “helping victims” (Gura and Roma, 2024, p. 52).

Moreover, various studies have shown that sports and other games foster relationships, collaboration, peaceful environments, and healthy co-existence (e.g., Schulenkorf, 2010; De San Eugenio et al., 2017; Sobotová et al., 2016; Brake and Misener, 2020; Gadais et al., 2023). For instance, using interview data, (Schulenkorf 2010) studied the outcomes of an intercommunity sport event in which 150 children participated, all from various ethnic backgrounds in Sri Lanka. The aim of this event was to strengthen relationships among Sinhalese, Tamils, Muslims, and Burghers who had faced historical conflicts that resulted in a civil war in 2008. Findings showed that many participants experienced social integration and reconciliation by forging new friendships, untangling stereotypes, and developing identities as peacemakers. Similarly, in Colombia, (Sobotová et al. 2016) evaluated the impact of *Tiempo de Juego* (Time for Play), a local non-profit organization program that provided daily training sessions in six different sports and art activities (e.g., soccer, basketball, cheerleading, breakdance) for almost a decade. In the context of the Colombian peace process, this program aimed to prevent youth in the community of Cazucá, Soacha from exerting influence on issues associated with violence and criminality (e.g., gangs, drugs, insecurity) given their high level of vulnerability. Cazucá, Soacha, was originally formed through informal settlements marked by high poverty rates and homes of families affected by internal displacement, violence exposure, and unemployment. Through participatory mapping, 35 young instructors (between the ages of 15 and 25) collectively described the program as affording safe spaces, a sense of community, gender equality through mixed-gender teams, and leadership roles for females. According to this research, the social change achieved in their studied communities cannot be measured only by the integration of sports, since other efforts have also contributed to this progress (Schulenkorf, 2010; Schulenkorf and Sugden, 2011; Sobotová et al., 2016). This caution accounts for the complexity of peacemaking processes.

In sum, growing research indicates that play has the potential to promote internal and social peace among child-victims of violence-related trauma. These findings, combined with the ongoing wars and political crises currently affecting various parts of the world, including Latin America, compel us to deepen our understanding of play as a powerful tool of peacebuilding, especially as this crisis is severely affecting the lives of displaced children, our most vulnerable population. In light of this, strengthening the evidence base is increasingly important. A key part of this scholarship is examining how the peace-building potential of play is experienced by children themselves, particularly those who experienced the trauma of violent conflicts and displacement in different international settings–an area to which our study contributes. It is worth mentioning that, although the importance of play therapy for children affected by conflict is well-established in psychological literature (cf. also Hyder, 2005; Cohen et al., 2010; Cohen, 2014; Humble et al., 2018; Cohen, 2019), the healing potential of children's everyday play in their natural settings (school, home, neighborhood) is less explored, especially in the context of displacement. Our study fills this gap and does so in a participative, child-friendly manner.

## Background

In Latin America, rates of violence have increased steadily over time. Political crises, organized crime, homicide, and femicide have spread fear into the daily lives of many Latin Americans, forcing an unprecedented displacement crisis in the region. According to UNHCR (2024), 42% of new asylum seekers in the world are from Latin America and the Caribbean countries. Venezuela, a country of origin for the majority of children in our study, is a prime example of this phenomenon. UNICEF reports that over 7 million people have fled Venezuela, resulting in one of the largest migratory crises in the world (Bueno, 2024, p. 4). Sadly, the political, socio-economic, and humanitarian crisis growing under the Maduro regime is directed also against children and adolescents, making them victims of imprisonment and torture for sharing anti-government content on social media (OCCRP, 2025).

In Chile, there has been a significant increase in the number of migrants and refugees over the past three years. The country ranks fourth in South America in terms of immigration, receiving a total of 1.6 million foreigners, mainly from Venezuela (32.8%), Peru (15.4%) and Colombia (11.7%) (OIM, 2025, p. 25). Within this migratory group, 13% are children and adolescents (OIM, 2023, p. 25). It is important to highlight the irregular situation faced by 65.9% of these migrants and refugees (OIM, 2023, p. 11), who arrived in Chile via illegal routes (one of which is the journey through the Atacama desert, where the temperature range is extremely high, reaching highs of 45 °C during the day and lows of −2 °C at night) and lacking the necessary documents to find legal work and provide a decent living for their families. Demographically speaking, the largest percentage of migrants (24%) resides in the Metropolitan Region (Servicio Nacional de Migraciones, 2023, p. 3). Within the Metropolitan Region, the communes of Santiago and Estación Central have the highest concentration of migrant inhabitants and are two of the communes with the highest rates of violence (Biblioteca del Congreso Nacional de Chile, 2024, p. 47–49). The school where this study took place is located in the Santiago district, where a multidimensional poverty rate, measuring deprivation in health, education, and standard of living, reaches 16.5% (Ministerio de Desarrollo Social y Familia, 2022). In addition, in 2023, the district rate of reported serious crimes—i.e., armed robberies, serious injuries, homicides and rapes– reached 3,749 cases per 100,000 inhabitants, exceeding the national average by 226% (Biblioteca del Congreso Nacional de Chile, 2024).

This data demonstrates that Chile, similarly to other Latin American countries, suffers from the continent-specific mix of criminal, social, and political violence that trickles down into many neighborhoods, schools, and even homes, taking a tragic toll on the youngest population as well. An example of this phenomenon is the “Tren de Aragua,” a criminal, transnational mega-gang originating in Venezuela but present in many countries in the region, including Chile, and seriously threatening the stability of the region through arms, drug and human trafficking, kidnapping for ransom, murder, and other crimes (Sampó and Troncoso, 2024, p. 152). Perhaps the worst of the effects of this endemic violence is the deprivation and criminalization of the children themselves. According to national statistics (Ministerio de Desarrollo Social y Familia, 2017), there are more than 500,000 “ninis” (adolescents from precarious socioeconomic backgrounds who are neither studying nor working) in Chile. These children are particularly at risk of falling victim to crimes such as human trafficking, or, worse, being recruited by criminal gangs and involved in their illegal activities.

## Our study

This is an exploratory, qualitative study designed to allow children to share their unique perspectives, experiences, and emotional worlds. The purpose of this study was to understand the function of play as a tool for supporting the mental recovery, social integration, and resilience of migrant and refugee children affected by political and social conflict. The research questions guiding this study were: (1) How does political and social violence experienced by child migrants and refugees affect their everyday life and wellbeing? (2) How does play function as a tool for the mental recovery and resilience of child victims of political and social conflict? (3) How can play-based interventions contribute to integration and peacebuilding for migrant children affected by political and social conflict? By responding to these research questions, we seek to promote awareness about the importance of children's right to play in the situation of political and social violence and to deepen the understanding about its role as an instrument for inner and social peace.

## Materials and methods

### Participants and ethical considerations

We implemented a number of strategies to protect children's rights, inclusivity, agency, and decision making, as well as to promote a collaborative relationship with researchers to decenter adult power (Swauger et al., 2017). In our workshops with children, we created a playful environment where there were no right or wrong answers. While children's parents provided consent to participate, we constantly sought assent from children. The principal investigator explained the nature, objectives, and procedures of the study to children in a simple, child-friendly manner. Children signed an assent form with their names and stickers. Children (as well as parents) were informed that they could withdraw from the study at any time. We also provided an option to temporarily withdraw and use a comfortable couch, located outside the play space in the corner of the library and labeled as the “Corner of Peace.” The protocol of child safety, which was observed throughout the study, specified that a child who retreated to the “Corner of Peace” would be automatically taken care of by a professional psychologist. The study was approved by the Ethics Committee for Social Sciences, Arts and Humanities of the Pontifical Catholic University of Chile (approval number: 231222003). The school director authorized the study.

In order to help the children feel safe, familiar and at ease, the study took place in the colorful space of the school library, where extracurricular activities usually take place. Our study included 23 children between 9 and 12 years old, from a variety of cultural backgrounds, and an equal ratio of girls to boys (see Table 1). The workshop was attended by migrant and refugee children of different nationalities: 11 children were from Venezuela, 10 children from Peru, 1 child from Bolivia, and 1 child from Ecuador. Interestingly, during the last session (framed as a “Children's Congress”), where young participants could represent a country or region of their choice, some children chose to represent their country of resettlement (Chile) or other countries in the world (for example, since children were informed that their actions and opinions are meant to help all children in the world, one child decided to represent war-torn Ukraine).

**Table 1:** Participant demographics.

**Characteristic**	**Number (total, *n* = 23)**
**Sex of child**	
Male	13
Female	10
**Nationality**	
Venezuela	11
Perú	10
Bolivia	1
Ecuador	1
**Age of child**	
9 or 11	16
11 or 12	7
**Grade levels**	
Third grade	5
Fourth grade	11
Sixth grade	7

### Data collection methods

The choice of study methodology considers children as subjects of rights (UNCRC, 1989), and therefore, subjects of research (as contrasted to be only objects of it), which in our study was reflected in positioning children as experts in play (and thus teachers to adults on the subject), as well as active participants of the research process (Kyritsi, 2019, p. 40). We collected our data in an urban public school located in Santiago, Chile. The study consisted of four play workshops entitled “Experts in play,” which combined a playtime and play-based investigation. As play is a fundamental form of children's participation in everyday life (Lester and Russell, 2010, p. 27), their language (Landreth, 2021), and a means of empowerment (Hyder, 2005), each research instrument had a playful, participatory and creative character (cf. Groundwater-Smith et al., 2015; Montreuil et al., 2021; Sevon et al., 2023), giving space to children's voices. Table 2 provides a summary of our workshops, their themes, guiding questions, and main play-based activities. These workshops were carried out within a span of three weeks, which allowed us to collect data using semi-structured focus group interviews, student work samples using drawing, and social cartography through maps (Rodríguez-Castrillón and Amador-Baquiro, 2023).

**Table 2:** Workshop overview.

**Workshops and themes**	**Discussion questions**	**Description**
Workshop 1 *Getting to know each other*	Who are we? How do we like to play?	Interactive explanation of this project and drawing children's favorite game while sharing why they like it and how often they play
Workshop 2 *The importance of play*	What is the importance of play in children's lives?	Children explored the importance of play through a question-and-answer dice game and a collaborative “Patchwork Quilt.”
Workshop 3 *Wellbeing of children*	How does play make children feel?	Using social cartography, children created two maps: one tracing their migration journey to Chile and another mapping their play spaces in Chile. Emotion cards helped them express feelings about these experiences.
Workshop 4 *Play and peace*	Can play help children live at peace with other people?	We explored children's views on play in integration, social peace, and wellbeing through a “Children's Congress” role play. Using photographs of conflict and peace, they discussed their definitions of these concepts and shared perspectives on social issues.

Our principal research instrument was a play-based focus group. We opted for this method because it allows a safe peer environment for children and helps to avoid power imbalances between researchers and participants, which can occur between an adult and a child in a one-on-one interview (Shaw et al., 2011; Adler et al., 2019). These focus group interviews were semi-structured, allowing a balance of structure and flexibility. This approach encourages rapport, topic shifts, and digressions based on the interviewee's interests (De Fina, 2020). To enable the participation of all children, also the more timid ones, we also invited them to respond either orally or to write down their personal opinions on the importance of play, concepts of violence and peace, etc. The second research method was drawing, which is known to provide access to children's emotional words (Jolley, 2010; Fargas-Malet et al., 2010). Drawing is also considered an enjoyable and playful activity for children and an important means of non-verbal communication for them (Longobardi et al., 2022). In order to find out the children's migration itineraries and experiences, we used a third instrument: mapping (Darbyshire et al., 2005; Groundwater-Smith et al., 2015), whereby we asked the children to draw their migration routes to Chile (and describe their experiences and emotions on the way).

### Data analysis

All types of material —transcripts, verbal productions, drawings and maps— are regarded as a single data unit and analyzed according to thematic analysis (Braun and Clarke, 2019, 2021; Naeem et al., 2023). The analysis is grounded in constructionist epistemology (Mogashoa, 2014) and combines deductive and inductive coding as well as theme development. The deductive codes reflect the study's main hypothesis that play can be an instrument of mental recovery, resilience, and reintegration, as suggested by the human-rights framework and a vast array of psychological and cultural theories of play. However, it is worth mentioning that this deductive approach was completed with the inductive theme development, as the children's unexpected openness about their migratory experiences, including the violence-related problems, provided very rich data on these themes. The generosity, political answers, and eagerness with which children talked about their difficult experiences (often initiating these subjects themselves) is a valuable finding in itself. It points to the need of migrant and refugee children to share their stories and be listened to in safe, welcoming settings.

The analysis followed a six-phase process, as outlined by (Braun and Clarke 2006). Specifically, the data was transcribed and anonymized by the research assistants who participated in the workshops, and then completed by a professional transcriber to assure accuracy. All transcripts were checked back against the audio recordings by the first author. Then, the authors applied (Saldaña 2015) first cycle coding, which involves an active, reciprocal engagement between our interpretations and the data. This initial coding phase followed a deductive and inductive approach, allowing the recognition of descriptive codes of our thematic analysis. To carry out this process, the first author examined all data from workshop transcripts as well as participants' drawings and maps, generating descriptive codes and an initial codebook. Using this codebook as a reference, each of us identified patterns across the data as an iterative process to carry out (Saldaña 2015) second cycle coding. Subsequently, codes were then collapsed or expanded collaboratively, creating a second codebook. Through an iterative process of reviewing, merging, mapping and renaming, the team developed themes and subthemes that aligned with our research questions. During this process, our themes and subthemes were then refined, discussed and conceptualized by the whole research team. To enhance trustworthiness, we applied Braun and Clarke's 15-point checklist of criteria for good thematic analysis to our study (Braun and Clarke, 2006).

It is worth mentioning that our data interpretation also includes the translation process of our participants' voices, who are native Spanish speakers. (Van Nes et al. 2010) highlight the challenges of translating participants' speech, particularly when culturally specific terms lack direct equivalents in another language. To minimize meaning loss and improve the validity of crosslinguistic qualitative research, Van Nes et al. suggest strategies such as collaborative translation and seeking expert input. In our study, we employed peer debriefing (Houghton et al., 2013) to collectively validate interpretations, using contextual clues from audio recordings. However, despite these efforts, fully capturing the depth of experience remains difficult, and some linguistic expressions may be untranslatable—an issue that is especially relevant when interpreting children's experiences through an adult lens.

## Results

The aim of this article is to explore the challenges faced by migrant and refugee children affected by political and social violence, as well as to examine the role of play in promoting mental recovery, resilience, and integration for these children. To achieve these objectives, we present our findings organized into five overarching themes, each with sub-themes, which are discussed in this section.

### Children's exposure to political and social violence

#### Political conflict and armed violence

Our analysis indicated that many of our participants' migration experiences were characterized by violence related to political conflict that affected children's wellbeing and mental health. These results match those observed in earlier studies evidencing severe, long-term mental health problems in migrant children, especially those who experienced armed conflict and other types of violence (Halevi et al., 2016; Shenoda et al., 2018; Jones-Mason et al., 2021; Bürgin et al., 2022). In this study, we found a pattern of political violence, social violence and economic instability that propelled children's families to seek safety by leaving their countries. The following excerpt illustrates how children are direct eyewitnesses and victims of political violence:

*B (boy): What happened in Caracas once—in Venezuela—we were doing elections to see who would become president, and Maduro won, and some trouble started*.

*G (girl): (interrupting) He won?*

*G: No, he cheated*.

*G: He cheated*.

*B: That is why tanks were taken to Caracas, and things like that. They exploded cars, and a young man was even taken to the hospital*.

*G: And Maduro also kidnaps children. He kidnaps people who don't agree with him and those who give their opinion on social media*.

*B: And makes them disappear*.

*G: This is why most Venezuelans leave for Chile, because of the elections, that's why*.

*B: To provide for their family*.

This excerpt illustrates how children become targets of violence amid political upheavals. Witnessing violent acts such as the kidnapping, car explosions, and enforced disappearances heightens their awareness of their own vulnerability and the threats this violence poses to their families' survival. At the same time, this awareness helps them make sense of their migration journey and identity as part of a national collective with the responsibility to *provide for their family*. Additionally, this passage challenges the notion that children are merely victims or passive bystanders of socio-political conflict. From an early age, migrant and refugee children engage actively in politics, conceptualizing their experiences and forming opinions that are far from neutral due to their direct or indirect involvement. This has significant implications, as child migrants have the potential to shape the political landscape of a hosting country in their adulthood (Humphries et al., 2013). Because our team shares this perspective of children as political actors, we introduced the concept of a Latin American Children's Congress during our fourth workshop. This initiative positioned participants as ambassadors tasked with proposing solutions to common problems.

While some children described Latin America in positive terms, highlighting its *good food, beautiful landscapes*, and *different types of animals*, many of their descriptions were strikingly negative and reflected deep-seated concerns:

*B: Latin America is dangerous*.

*A (adult): Why?*

*B: Because they steal a lot*.

*B: There are assaults*.

*G: Crime*.

*B: Mafias*.

*G: Violence*.

*G: Animal abuse*.

*B: Child abuse*.

*B: Mexican cartels*.

*A: What does “child abuse” mean?*

*G: When children are beaten*.

*B: Miss, Venezuelans even more, because we live…we practically grew up in these conditions*.

Even if children in our study did not experience armed conflict (e.g., war, non-international armed conflict) they did experience armed violence, which UNICEF (Gregson, 2024, p. 5) defined as “the intentional, threatened or actual use of arms to inflict death or injury—takes many forms, ranging from political to criminal to interpersonal violence.” As one Venezuelan child explained:

*B: All parts of Venezuela are dangerous, but it's safer in Maracaibo because Caracas is pure lead*.

*A: Pure what?*

*B: Pure gunfire*.

Our young participants' perception of their cultural region encompasses a sense of unsafety and danger to their childhood, in which violence is prevalent. Children's perception is shaped by their lived experiences in their home country, and we argue, directly related to their migration experiences. Overwhelmingly, studies have demonstrated that migrant children face a heightened risk of exposure to violence and the associated psychological distress stemming from their experiences before, during, and after migration (Barajas-Gonzalez et al., 2018; Jolie et al., 2021).

Besides violence, escaping from poverty was another reason for migration that was emphasized by many of our young participants. When a researcher asked about their family, for instance, a boy stated: *They are all in Venezuela… they are poor, that is the hard reality*. Children's remarks reflect not only the economic struggles that force migrant parents and children to leave their home countries, but children's sense of helplessness resulting from long-term separation with their families and the emotional impact that this *hard reality* has on children's lives.

Research has shown that the stress of migration coupled with family separation increases the risk of life-long mental health problems, including depression, anxiety, substance use, and suicidality (Wallace, 2024).

In our study, our participants also shared the impact that political violence and instability have on their mental health:

*A: How do you feel in those situations?*

*G****:*** *Sad, because in Venezuela there is too much violence because of a corrupt president, who is very bad*.

*G: Bad, because I am a person that when I see somebody crying, I also feel like crying*.

*G: Depressed*.

*G: Sadness too, because that will also happen to us any time soon*

*G: Bad, in pain, and feeling sorry*.

*B: Anguished because we never know when other people's hard experiences are going to happen to us too*.

In this excerpt, we can observe that violence takes a toll on children's psyche. Our participants often identified violence as a product of the political management in their country. This was especially true for children born in Venezuela. Also, we found a connection between children's negative feelings with an anticipated distress about a future, as reported by children. This finding is consistent with previous research demonstrating that migrant children's anxiety levels and post-traumatic stress symptoms are significantly higher due to warfare exposure (Celik et al., 2019; Pumariega et al., 2022).

Armed conflict and political violence in the country of origin are usually followed by a perilous journey to safety, which further affects children's wellbeing. As children reported:

*B: [The trip was] difficult and good at the same time*.

*A: Why?*

*B: It was mostly difficult on the bus, because we were always stopped by the police…and when we started to walk through the desert, we had to run away when we saw the police*.

*A: Oh, the police. That must have been stressful*.

*B: Yes, they would grab me, and they were going to take me back*.

**Figure 1:**
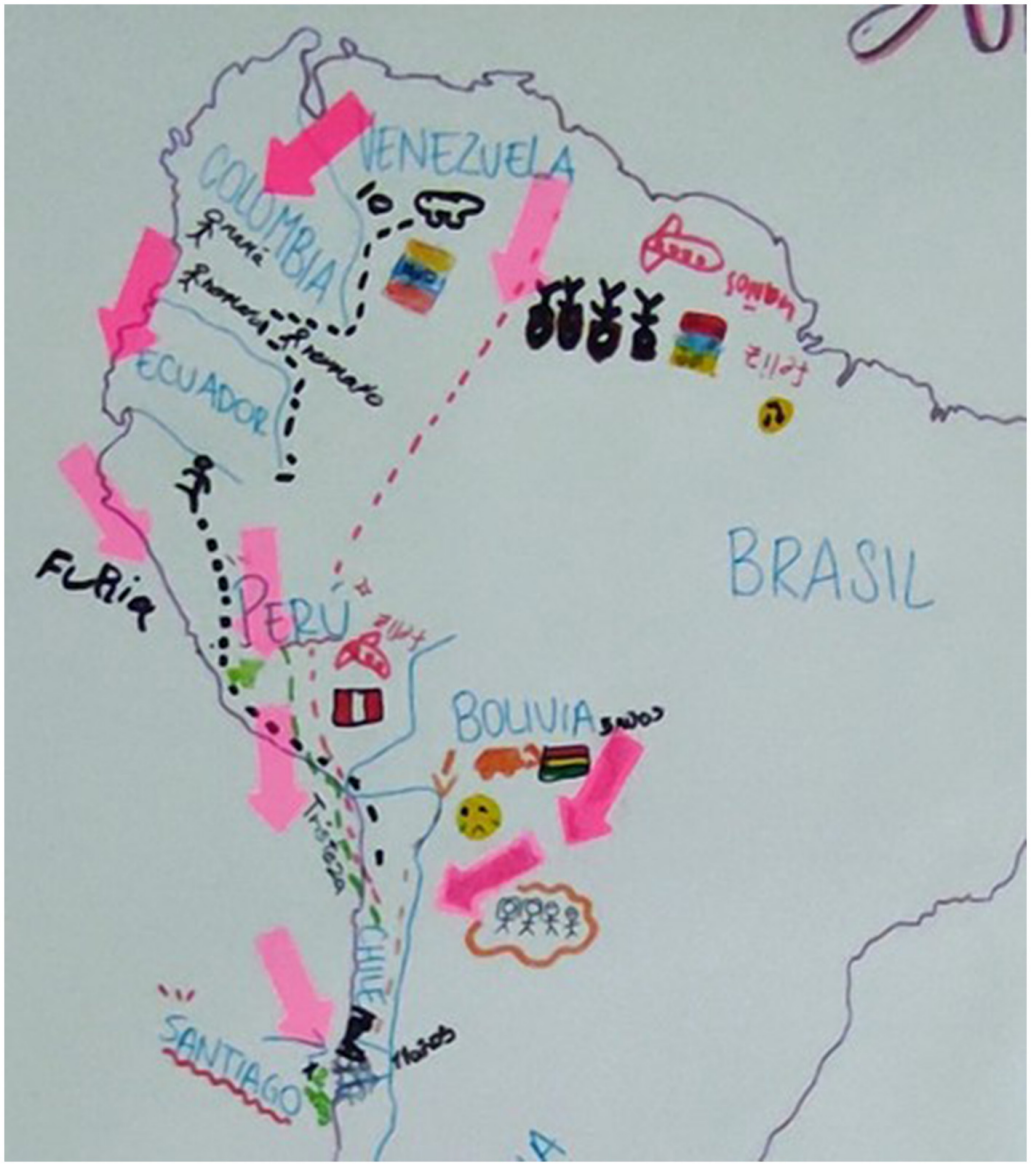
Map with migration trajectory.

This excerpt describes the kind of experiences that our participants had to endure during their migration trajectories. Most of our participants endured long journeys across various countries (with some doing at least part of the journey on foot through the Atacama Desert). One of the workshops included the drawing of participants' journeys on a map. Figure 1 shows the immigration journey of Venezuelan children and their families. One child describes feelings during this trajectory as *furia* [*anger*], albeit his stay in Peru was expressed as *feliz* [*happy*], and his journey turned into *tristeza* [*sadness*] with a stop in Bolivia, to later move to Santiago, Chile. This map reveals a long transition process involving a geo-temporal and emotional trajectory of more than a year of migration. According to this drawing, during this process, our participant experienced a high level of instability and disruptions, at least in terms of family configuration, residence, emotional distress, and physical burdens. As presented in this map, migrants reterritorialize the map by creating a new cartography of the region shaped by emotions and experiences. They develop affective bonds of connection and disconnection with the territory, depending on various factors. This finding further challenges the narrow notion of migration as a linear movement that ignores the complex realities associated with migration decision-making, including individual, cultural, economic, political, interpersonal, and linguistic considerations (Lu et al., 2020).

#### Social violence and discrimination toward child migrant and refugees

Unfortunately, the violence-related trauma does not end with fleeing countries in crisis. All over the world, child migrants and refugees experience discrimination and other forms of social violence in countries of resettlement, which further jeopardizes their emotional and psychosocial wellbeing (Metzner et al., 2022). In our study children reported many experiences of direct or indirect social violence in different spaces of their everyday life (e.g., school, social space, public transport):

*B: When I came to the workshop, there was a Chilean boy, so when he had the ball, I would come up to him like this, so he got annoyed and insulted me and at one point he got fed up and hit me in the guts*.

*B: The other day, on Saturday I went cycling and I was riding my bike and a boy pushed me and he gave me the finger*.

*A: And what do you think is the reason for that? Do you think it's because you're foreigners or just because Chilean children are more aggressive?*

*B: Because they think they have more authority because they are from this country, and not from another one*.

Children also become witness to and victims of adult conflicts that arise on the background of international animosities between migrant and local communities:

*B: Once I was traveling because I was coming to the soccer practice on my own that day. I got on the bus and there was me, a lady and the driver. The driver was Venezuelan and the lady was Chilean and she was saying a lot of things to the driver because he wouldn't open the door for her because it was in the middle of the road and the lady wanted to get out and they started to fight, and I almost broke my hand because he braked so suddenly and I had to hold on*.

As it is well evidenced in literature, social violence affects children's mental health, leading to depressive symptoms, decreased self-esteem, adaptation problems, and lower school achievement (Guerra et al., 2003; Espinosa, 2020; Oczlon et al., 2021).

### Children's conceptualizations of violence and peace

**Figure 2:**
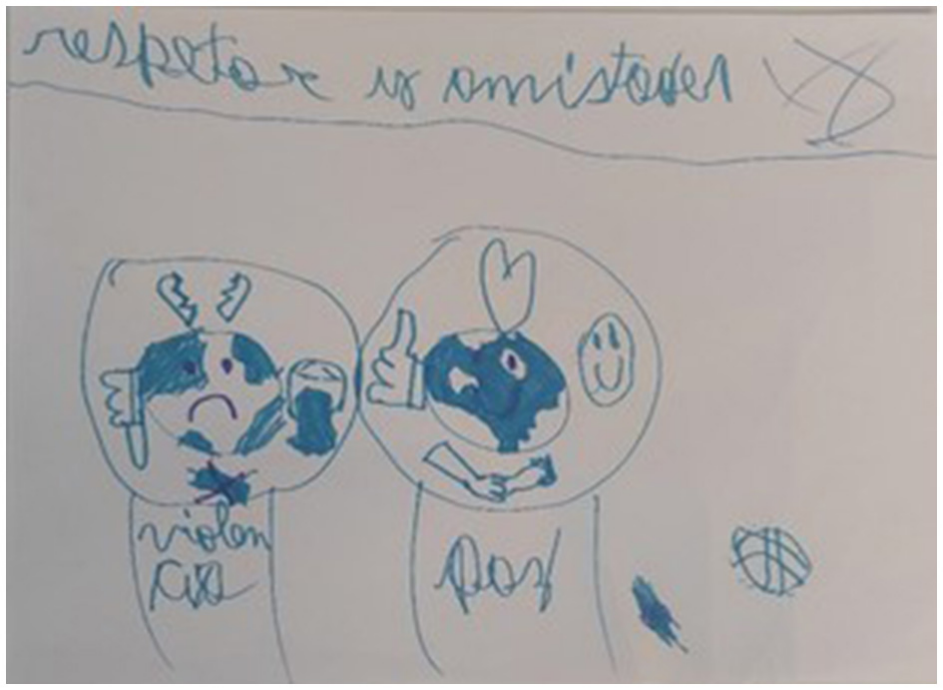
Respect and friendships. Violence and peace.

In our study, children not only recalled their experiences of violence but also explained how they understand and conceptualize violence and peace, and how both phenomena make them feel. Figure 2 gives a glimpse of the happiness that children experience in peaceful social conditions, and the sadness that is provoked by violence and conflict.

Children's definitions of violence are not abstract constructs, but generalizations of their personal experiences, in which the various dimensions of violence –political, and especially social and interpersonal– intertwine and influence one another.

*A: What is violence to you?*

*G: Mmm... fights, at school, in the streets or, I don't know, anywhere*.

*G: Murder attempt*.

*G: Humiliation at school*.

*B: Bullying*.

*(…)*

*G: For me, it's just being hit, pushed, insulted, threatened or shouted at. That's it*.

*A: Have these things happened to you?*

*G: Yes*.

*A: And how do you feel when these things happen?*

*G: Sad and alone*.

#### Negative peace

The majority of children defined peace in negative terms as the absence of conflict, both on the political, social and interpersonal levels:

*A: And what will peace be for you?*

*G: Non-violence, tranquility without the need for weapons or violence*.

*(…)*

*A: Does anyone else want to share what they understand by peace?*

*B: Me! Playing without fighting*.

*(…)*

*G: Peace is when there is no violence or abuse of people or children*.

*B: Peace is when there are no kidnappings and there is no danger*.

#### Positive peace

Interestingly, many children in our study also captured peace in positive and active terms:

*A: What does peace mean to you?*

*B: Peace is making friends*.

*G: Peace is when people help each other and live together, it's like saying that they live in a healthy environment all together*.

*B: For me it's when you're with a family member or a friend*.

*B: Peace is when you're in your safe place*.

*G: Practicing harmony because we know there won't be any more violence*.

*B: Solve problems by talking, not by hitting or threatening*.

**Figure 3:**
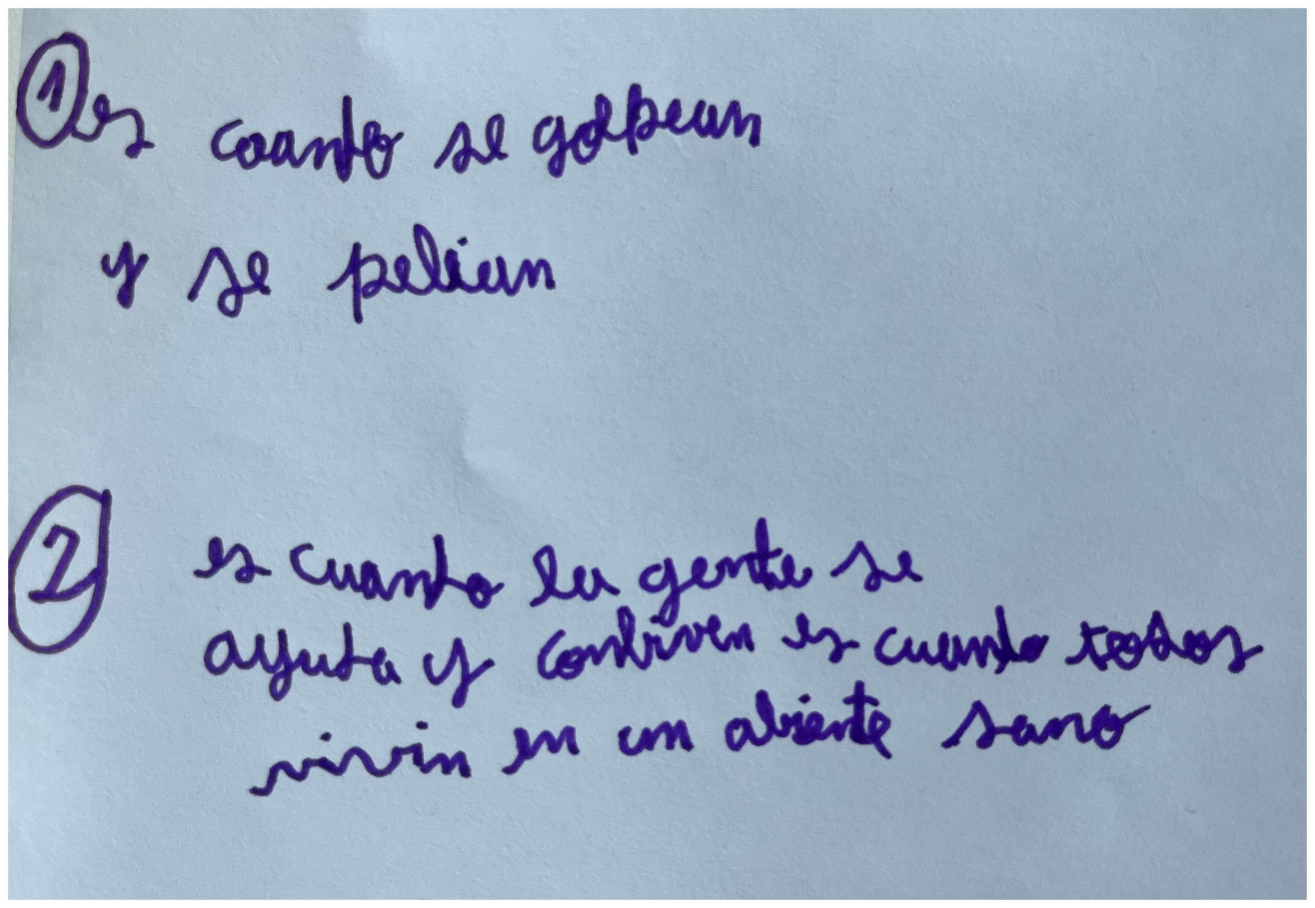
[Violence] is when they hit each other and fight. 2. [Peace] is when people help each other and live in harmony, and when everybody lives in a healthy environment.

The children intuitively understood that peace is something more than only the absence of weapons and direct violence and astutely distinguished its various, actively practiced dimensions such as friendship, social harmony, solidarity and mutual aid, security, and peaceful conflict management. This corresponds to the theory of positive peace, developed by (Galtung 1969), the very founder of the discipline of peace and conflict studies. As he classically argued, the transformation of our conflict-torn world will not happen through a ceasefire alone but requires a continuous and determined pursuit of positive peace. When describing his theory of positive peace, Galtung used the metaphor of health, arguing that “health can be seen as the absence of disease” or, in positive terms, as “the building of a healthy body capable of resisting diseases, relying on its own health forces or health sources” (Galtung, 1985, p. 145). In a similar way, peace can be negative when it is perceived as an “absence of violence” in its direct form or as a positive striving toward social justice, solidarity, and civic friendship, requiring a practice of non-violent conflict resolution of many disagreements and tensions that inevitably reappear in our social lives. It is remarkable that, similarly to (Galtung 1985), one girl used the metaphor of health (*healthy environment*), to describe the peaceful state of interpersonal relationships (also displayed in Figure 3).

#### Inner peace

Interestingly, some children also understood peace in a more intimate way, referring to it as an interior state: “peace of mind” or “inner peace”.

*B: Peace is being calm*.

*G: Peace is when I'm with myself and when I'm calm*.

*G: For me, peace is when it's night-time and everything is quiet and dark*.

*B: When there is peace and harmony*.

*B: Peace is being calm*.

*G: Being relaxed and calm*.

Many scholars have argued that peaceful relations in the external sphere begin with the personal non-violent attitude (Kool, 2008), cultivation of inner peace (Redekop, 2014), and personal peacefulness (Nelson, 2014). However, peace psychology is a very young discipline (e.g. APA Division 48 created in 1990 [cf. APA, 1990]), within which very little attention has been paid to children (Balvin and Christie, 2020). The concept of children's inner peace, its components, mechanisms, and factors contributing to its development are therefore still unknown. As (Guetta 2020, p. 248) observed, peace education programs, if existent, focus on external behavioral or communicative factors with little room left for investigating the inner driving force that motivates people toward peaceful practices or actions. Against this background, our study demonstrates that children do distinguish the inner component of peace, the study of which could be an important contribution to peace education, especially in a context of high prevalence of external—political, social and interpersonal—violence.

### The importance of children's rights in children's views

Our study, which adopted a rights-based approach to children's play, in its course gathered children's own opinion about the importance of their rights, and their meaning for peace, which we will report on in this section.

Article 42 of the UN Convention on Children's Rights obliges state parties to “make the principles and provisions of the Convention widely known, by appropriate and active means, to adults and children alike.” This study demonstrates that play and playful investigation (which, by consulting children, recognizes the importance of their voices and opinions) can be effective means of fulfilling these obligations. In the introduction to the study, the principal investigator explained to the children that play is their right, “inscribed in important international documents.” The children then understood the concept of having rights, and very quickly extrapolated that they were subject to other rights, as well, which can be seen in the following dialogue:

*A: What children's rights do you know?*

*B: The right to play, the right to play, the right to eat, the right to breathe*.

*G: The right to have more friends*.

*B: The right to have a home*.

*B: The right to go to school*.

*G: The right to have a family*.

*G: The right to speak*.

**Figure 4:**
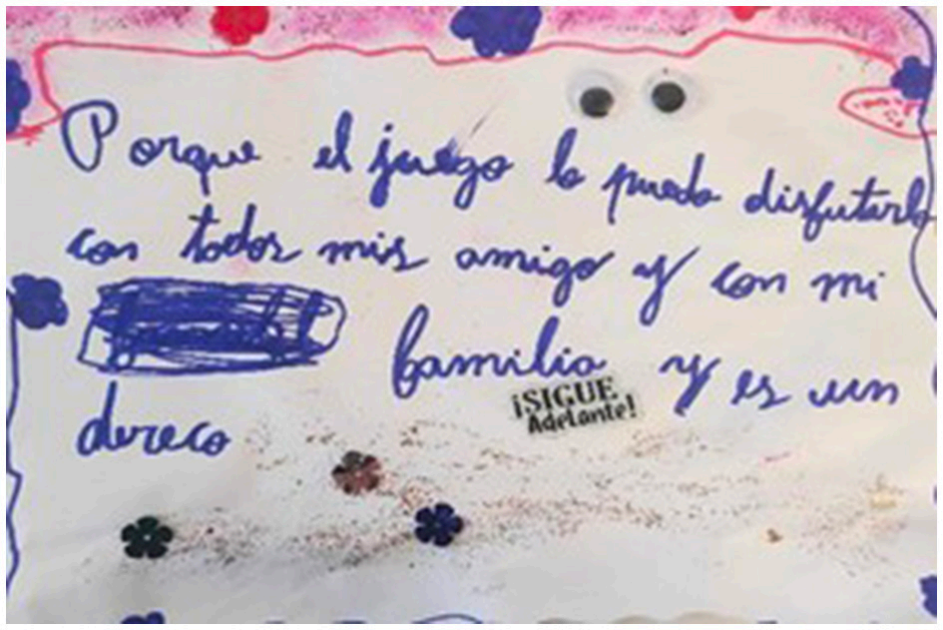
[I like playing] because I can enjoy it with all my friends and my family, and it is a right.

The newly acquired awareness that play is not only their favorite activity but also their right empowered the children (Figure 4). The following dialogue illustrates both the empowering potential of human rights education and the importance of play in children's lives:

*A: How would you feel if, for example, your parents or your school or the president of Chile told you “Children can't play any more”?*

*G: Well, I'd punch him*.

*[laughter]*

*G: I'd still play, because it's a right*.

*B: I'd take them to jail*.

*B: I'd leave the country then*.

*A: Antonio [child's name changed], were you going to say something?*

*B: I would be sad*.

*A: You would be sad, why?*

*B: Because I want to play*.

*(…)*

*B: I would protest*.

*B: I would go back to my country Peru and that's it. We're all free*.

#### Children's rights for peace

Importantly, during the Children's Congress session, children were asked to decide which of their rights can contribute to creating a more peaceful world.

*A: What children's rights can help us live in peace with other people?*

*B: Playing and having fun with friends... and playing Free Fire or Roblox*.

*G: Children's rights that can help us live in peace are giving our opinion on, for example, child abuse, women abuse..*.

*G: For me, children's rights are being able to play, have fun, eat, sleep and... play again*.

*G: For me, the most important thing is not to hit each other, to respect each other and to be with other people without harassing or bothering them because that's really ugly and you can't live in peace like that*.

*G: I think respect, playing and having fun, being with the people who love you, being with your friends and... that's it*.

*G: Helping us to live in peace with play... for everyone's peace of mind*.

*G: For me, the rights of children that help us live better are to play, to live in harmony, to do activities together*.

*B: The right to participate*.

*A: In what way?*

*B: Like I'm doing right now*.

*G: To live in peace, it's [inaudible] to meet more people and live in harmony with the world and also to learn*.

It is worth mentioning that this dialogue illustrates quite well the children's empowerment that took place during [our] workshops. In the first session, only the most self-confident children expressed their opinions, while in the last, even the timid and tacit ones actively participated, and as reported above, appreciated it. As one girl later summed up: *Sometimes adults only listen to each other, and don't consider children's words to be valid*.

When asked how the right to play contributes to peace, children explained it in the following way:

*A: Why is the right to play and have fun so important?*

*B: Because it can relieve stress*.

*G: The right to play can help us because we are all playing together, in harmony, in peace and without fighting*.

*G: We relax and feel good*.

*B: Relax the mind*.

*B: Distract the mind*.

*B: Because we play in a team*.

It is remarkable that, similarly to the Committee on the Rights of the Child (CRC, 2013, p. 31), which recognized the potential of play in physical and physiological recovery, as well as in the social reintegration of child victims of violence, children in our study identified the same two ways in which the right to play contributes to peace: through improving personal wellbeing (*relieving the stress, relaxing, distracting the mind*) and social integration (*playing together, in a team, in harmony*). Both aspects will be discussed in more detail below.

### The importance of play for inner peace

In this section, we present and interpret children's views on the importance of play for their well-being, and in particular for their mental health, which contributes to building inner peace.

#### Play and children's holistic well-being

One of the first conversations that we had with children was the following:

*A: Would it be important for children to play?*

*B: Yes [in unison]*.

*B: Very important*.

*A: Why?*

*G: Because otherwise you get bored and become sick*.

*B: You become an otaku [freak]*.

*G: Because if you don't, you have nothing to do all day*.

*G: For entertainment*.

*G: For health*.

*B: To have fun*.

*G: It's important to play for children because otherwise you get bored, and you'll never have a childhood*.

*G: To develop a skill*.

Children understand very well the intrinsic value of play for the dignity and pleasure of their childhood. In the response that without play they would *never have a childhood* one can hear echoes of the famous statement by (Van Gils 2007, p. 18) that play is “a child's right to be a child.” It is with astonishing clarity that children are aware that play is important not only *for fun* but has countless instrumental benefits for their wellbeing, cognitive (*developing a skill, learning*, or *neuron*s, as they later put it), and social development (*playing in a team, knowing more people, making friends*).

It was also very striking that the first thing that children mentioned, next to *not being bored*, is the importance of play for their health, which has only recently been recognized in literature (Milteer et al., 2011; Yogman et al., 2018; Tonkin and Whitaker, 2019; Nielsen, 2021). When asked to explain this health-related impact children said the following thing:

*A: Carmen [name changed], you said something interesting: “that children get sick when they don't play”; why do you think that happens?*

*B: Because they get stressed*.

*G: Because, because when you don't play you can get sick because of anxiety, being all day with nothing to do*.

*G: Professor, you can also get sick from being locked up all day and being all day with the people you already know and not getting to know more people*.

*G: Because being bored for so long makes you stir-crazy*.

*B: If we are locked up for so long, well, we can get stressed, bored and only meet one person in life*.

It is interesting to notice how well children understand the multifaceted importance of play for their health in its various dimensions: physical (*not being locked up*), social (*meeting more than only one person in life*), and mental. It is quite remarkable that the children assumed in an intuitive way a broad concept of health that means more than the absence of disease or malfunctioning of the body, but rather, corresponding to the holistic model of health developed in WHO documents World Health Organization (1946, p. 100) and medical literature (Schramme, 2023).

#### Play and children's mental health

Undoubtedly, however, within this multifaceted positive impact of play on health, children put the strongest emphasis on the importance of play for mental health. Although scarcely researched in the context of armed conflict, political violence and migration, play, as in this study, has been found to be “the most-used coping mechanism by children for dealing with extraordinary stress” (Chatterjee, 2018, p. 130). From a rights-based perspective, children, while playing, become agents who take active part in regulating their own emotions, navigating the stressful situation of violence or displacement, and adapting to life changes. Conversely, children who are denied the opportunity to play in situations of crisis may struggle in social interactions and develop problem-solving skills (Capurso and Pazzagli, 2016; Chatterjee, 2018). This finding merits an expansive view of the relationship between play and mental health.

**Figure 5:**
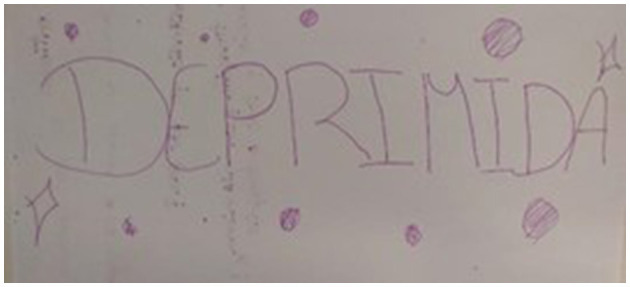
Depressed.

Throughout the workshop, the importance of play for mental health was repeated most frequently (fifty-three times), indicating the powerful contribution of play to mental health experienced by children in their daily lives. Indirectly, this also points to many stressful situations and mental health issues that they struggle with, such as *anxiety, stress, and depression* (see Figure 5).

On the basis of the children's reports, we were able to distinguish three main channels of the positive influence of play on mental health. The most direct channel of this influence is all the positive affect, joy, fun, and happiness that play produces, which for children living in difficult, precarious conditions cannot be overvalued. While playing, children enjoy the agility of their bodies (are not *locked up*), laugh with their friends and jump about excitedly. By boosting positive affect, play decreases mental distress, produces patterns of thought that are more flexible, creative and open to the environment, and thereby, leads to better coping (Pressman et al., 2019).

*A: How do you feel when you play?*

*B: When I play, I play with my mates, I play well, I have a great time, we play, we laugh... I have a lot of fun*.

In this context, a significant finding we identified in a few of our participants' migration trajectories was the fact that play positively influenced the long, dangerous, and difficult process of leaving home. For instance, one child explained, referring to his migration journey:

*B: I played looking at the landscape*.

*A: How so?*

*B: For example, I counted the cars that passed by, and I saw how beautiful the landscape was*.

*B: Yes, I played too*.

**Figure 6:**
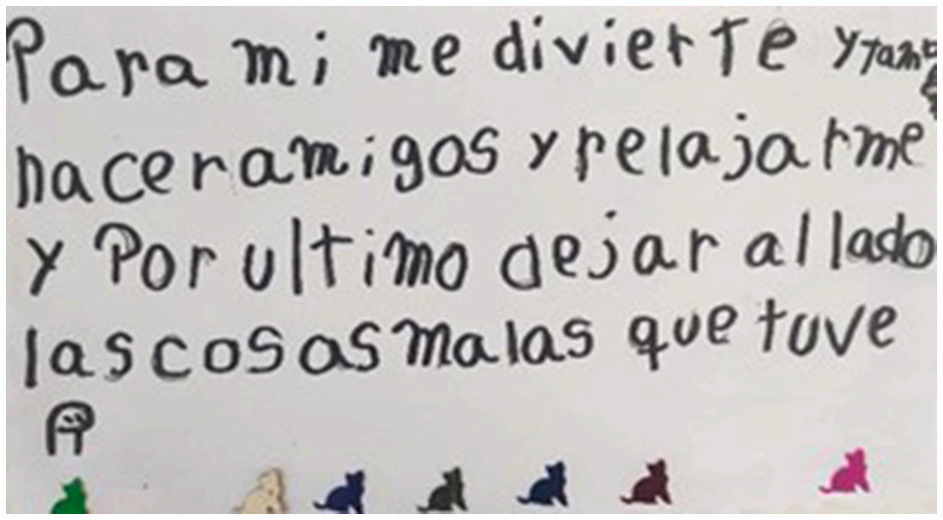
For me [play] is for fun and to make friends and relax and finally to let go of the bad things I had.

The positive impact of play on children's mental health is, however, a deeper and more complex phenomenon. There is consistent evidence that play increases resilience to stress (Jackson, 2006; Chatterjee, 2018), which was confirmed by the children who repeatedly said that play *de-stresses* or makes them feel *less stressed* and helps them to *relax* (as illustrated on Figure 6). The opinions of the children who participated in our study are congruent with what is reported in the literature regarding the positive influence of active play, exercise and sport on health and wellbeing (Hosker et al., 2019; Faigenbaum and Bruno, 2020).

**Figure 7:**
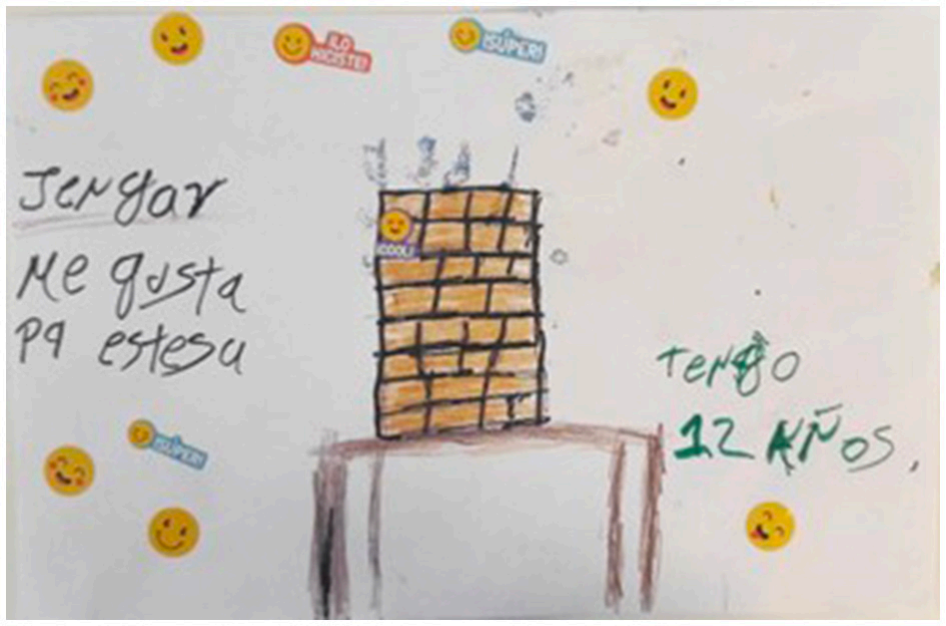
Jenga. I like it because it stresses me out. I am twelve years old.

Interestingly, for the task of drawing their favorite games, one child drew a game called *Jenga*, and then explained that he likes to play it because it *stresses him out* (see Figure 7). This can be explained well by Sutton-Smith's (2003) theory of play being a “parody of emotional vulnerability”. Within this theory, building resilience to stress is achieved through safe “as if” frames (Lester and Russell, 2010) of play that allow children to elicit and experience certain troubling emotions (such as fear, anger, stress, hate), without fully suffering their acuity and consequences. In this way, play gives the child an opportunity to master those emotions which could otherwise overwhelm a child with more sophisticated emotions and cognitive states such as “performance strategy, courage, resilience, imagination, sociability and charisma” (Sutton-Smith, 2003, p. 13). Handling stress in playful conditions may contribute to seeing future hardships as less intimidating and coping better with them in real life.

**Figure 8:**
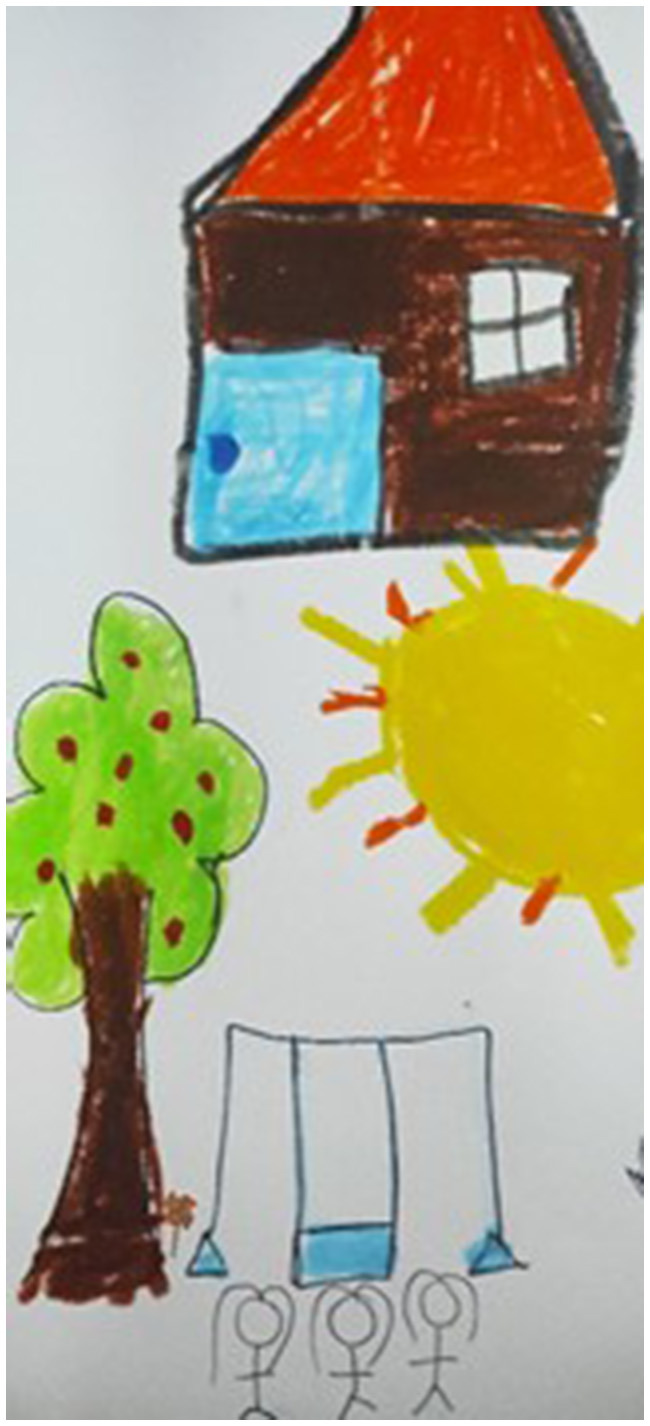
Place of play.

**Figure 9:**
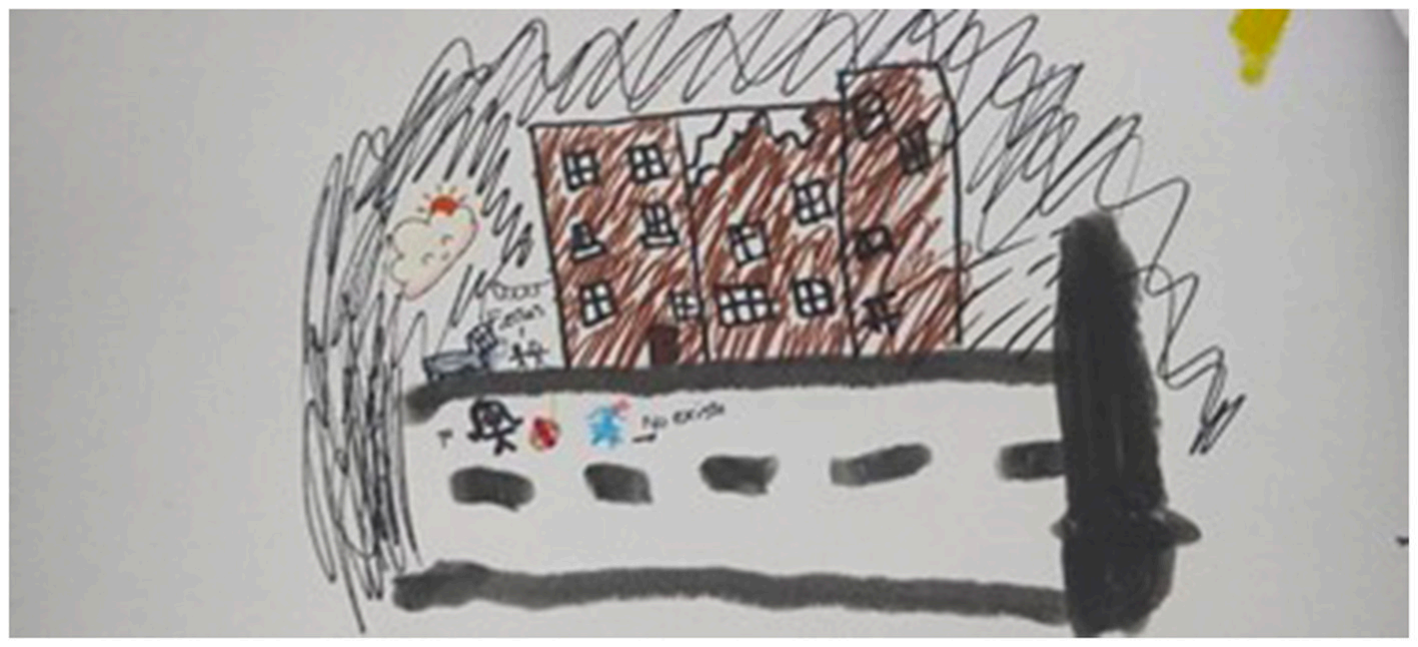
Place where play is lacking. Parties. Doesn't exist.

Moreover, thanks to its “as if” character, play also helps children to construct an alternative, safe and child-owned space, a space free of the problems and stressors of an adult-governed world (see Figure 8), while places where play was lacking were depicted as grim and dark (see Figure 9). This aspect of play as a child's safe island in the troubled waters of everyday life came up in many statements, where children reported that play helps them *forget their problems*, reminds them of *good times* or, and this was constantly repeated, *distracts their minds*. The children explained as follows:

*A: Ah, there is something that really strikes me in your answers, “distracting the mind.” What is that?*

*G: It's when you think of something that you would like to do, or that you like to do, and that could also be that it distracts you through play*.

*B: Playing with friends, and in a group and not fighting and living in peace*.

*B: To forget about problems*.

*G: It's like when you distract your mind with your friends, playing*.

*G: Distracting the mind is when we are bored, and we do something we like to do*.

*B: It's when you have a bad thought in your head and you want to get it out of your head, you can do it by playing or talking to someone else*.

*G: Distracting the mind is when we are in a happy moment, or it is also when we... forget the problems we may have with other people*.

### The role of play in social peacebuilding: potential and limitations

In this section we report and interpret the children's opinions on the importance of play for social peacebuilding.

#### Team spirit: integration and inclusion through play

**Figure 10:**
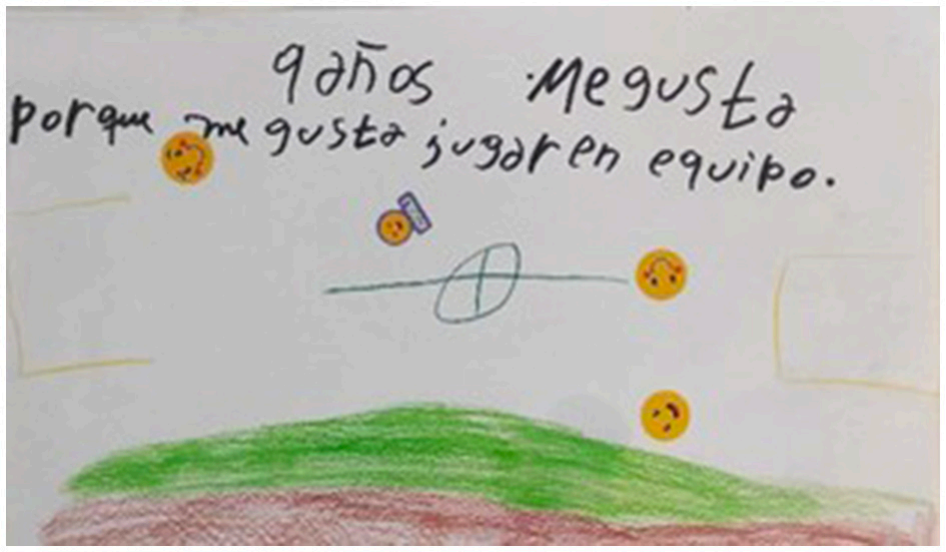
Nine years old. I like it because I like playing in a team.

One of the main joys of play is sharing time with others: friends, family and, as many children mentioned, animals. The recurring motto was *playing in a team*, which children repeatedly cited as the primary reason why they like the games they like (as illustrated on Figure 10), or as an explanation why they make new friends through play, or else as a factor that makes them *feel better, trust more* and *feel at peace*.

It is noteworthy that this team spirit was also extended to newcomers, making play an important instrument of inclusion:

*A: And if a child arrives from another country, can play help us get to know them better?*

*G: I think so, because once, in third grade, a boy from Brazil arrived, and I met him while playing spin the bottle. I was alone, and some girls invited me to play*.

#### Play and friendship

It would be difficult to overvalue the importance of play for friendship. First, play was mentioned as a main mechanism of making friends.

*A: Can play help us make new friends?*

*B: Yes, because we can play as a team, and we make new friends and all that*.

**Figure 11:**
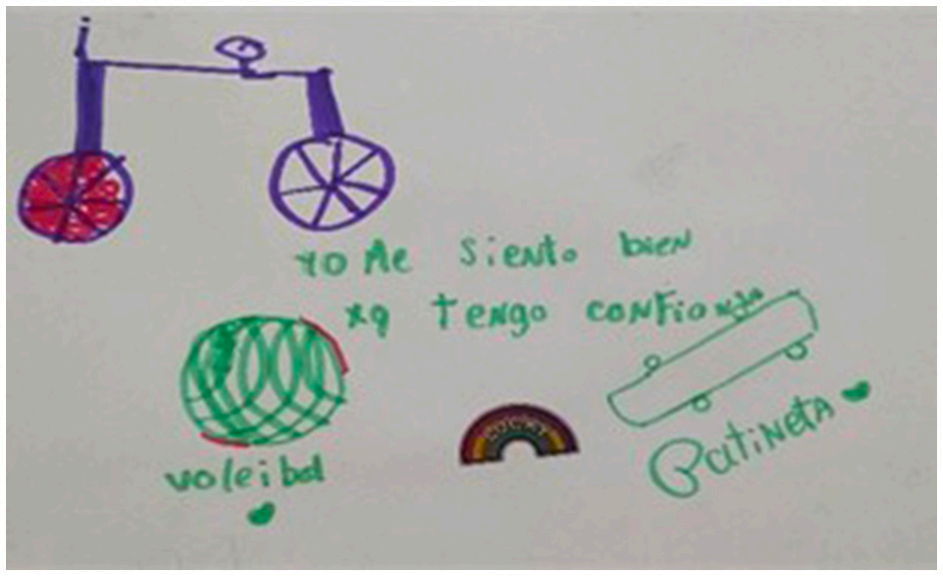
” [When I play] I feel good, because I trust. Handball, scooter.

Children explained that while playing, they *get to know more people, know them better* and *learn to trust them* (also reported by another child on Figure 11). As one girl astutely explained, while playing *you spend a lot of time with a person, you start to feel that this person is safe to be with and you start to feel trust toward them*.

For children, play is the substance of friendship. Playing together is simply what friends do and how they spend time with one another: *My best friend is called Sof*í*a, she's the one I like to play with, and... we just chat, hold hands and walk and chat, and that's it*.

Importantly, play in friendship is “for better and for worse.” Many children shared with us their experiences of grief, which often was aggravated by migration-related separation and the inability to say goodbye to a dying relative (e.g., a grandparent). Play turned out to be a means through which friends help each other in difficulties. As one girl recalled:

*G: I was absent because my dad had passed away and I was very sad. Then my friend told me to go out during break and play; then I started to feel better*.

It is important to mention that for these migrant children who at a very young age experienced the pain of separation and displacement, friendship is something they value very highly, something that, just as play, defines their childhood. One boy, when asked whether friendship is important for him said:

*B: Yes, extremely important, because it's something I have in my childhood, that I have to have in my childhood*.

Another child added:

*G: Yes, because they make us happy, they make us feel good... when they are with us*.

#### Play as a bridge between different cultures

Play has been described as a language of children. For example, (Landreth 1978, 2021, 2023) classically argued that it is through play that children communicate, express their feelings and form meaningful relationships with others. An important aspect of play as language is that it can “create bridges” (cf. Marsh, 2016) between children of different cultures, countries and mother tongues.

*A: Felipe [name changed], you said that you played with a child from another country even though you didn't speak the same language, is that right? Can you tell us more about it?*

*B: Yes, a Brazilian*.

*A: So, he was a Brazilian. And how was your experience?*

*B: I didn't understand anything*.

*A: But was the game good?*

*B: Yes, because we played Free Fire. Excellent*.

As children reported, play can easily become a bridge between different cultures because many games are international: among them, football, skate, hide-and-seek, or a marble game (which in different versions of Spanish different only in name, being called *canicas* in Venezuela, *bolitas* in Chile and *metras* in Colombia). This is perhaps one of the reasons why, as one child put it, *playing helps you feel at home*. The universality of play was also highlighted in the following dialogue:

*A: If a child comes from another country, can playing help them make friends?*

*B: Yes*.

*A: Why?*

*B: Because it doesn't matter what country you're from, where you're from, you're still going to play*.

#### Play and moral development

Play not only creates bridges and inclusive communities but also communities governed by rules and values. For children, play is an entry to the world of normativity, which children in our study understood very well in saying that what they learn through play are *new things and new rules*. As (Piaget 1965; cf. Gray, 2013) classically noticed, even a simple marble game is a complex social institution having an intrinsic code of rules and its own jurisprudence. As the author explained: “The little boys who are beginning to play are gradually trained by the older ones in respect for the law; and in any case they aspire from their hearts to the virtue, supremely characteristic of human dignity, which consists in making a correct use of the customary practices of a game. (…) If this is not “morality”, then where does morality begin?” (Piaget, 1965, p. 2). This inherent morality of play gives rise to one of its most important social lessons—that of fair play. Fairness, a constitutive element of good play, is, however, not the only value that children learn through play. Beyond trust, inclusion, and the intercultural dialogue mentioned before, children also mentioned learning *respect, patience in waiting your turn*, and *non-discrimination*.

*A: How do we treat each other?*

*G: Well, with respect, not offending each other, not discriminating against each other, for example if you, let's imagine I'm dark-skinned and you're white, then I say don't say she's white and not dark-skinned*.

Beyond understanding and respecting the rules, play, which is perhaps more important for the peaceful democratic community of today, also requires negotiating, modifying them, and resolving conflicts when the rules are broken (cf. Gray, 2013). One girl explained the potential of play for peaceful conflict resolution:

*G: More than anything, in my class, they fight a lot, they push each other, they pull each other's hair*.

*A: And can play help children who are always fighting?*

*G: Yes! Because they would get to know each other better and wouldn't have conflicts*.

#### Violence in play and playful conflict resolution

However, despite the unquestionably positive potential of play for social peace, children also experience violence and conflict in play. Our respondents reported numerous forms of aggression such as fights, cheating, mocking, and rejection.

*G: (...) It's bad with certain classmates, because most of them, if they lose, they push you, or they shout*.

*A: Ok and what do you think happens to those classmates who push when they lose?*

*B: They're envious*.

*B: They're frustrated*.

*A: They're frustrated? OK*.

*B: They don't know how to lose*.

*A: It's not that easy to lose*.

*B: It's easy*.

*A: It's easy? I find it hard*.

*G: Once we were in PE and we were playing with a big inflatable ball, and then M (girl's name) came and threw it at me and broke my arm*.

*G: One day I was playing, and this boy didn't know how to lose, so he got really angry, then he sent a friend to hit me, so I left because I didn't want any trouble*.

*G: I want to talk, but this isn't a story about friendship and peace, it's something else*.

*A: Ok, excellent*.

*G: On my birthday they took us out into the playground, and we were playing football and one of the boys, I don't know why he did it, I was just told that he'd done it. The two of them were pushing each other and just as I turned around, one of the boys kicked the other one*.

Children repeated similar stories on various occasions, clearly pointing to the problem of in-group aggression at school. As one boy reported: *I have a friend who, even though we treat him well, treats us badly. Because he insults us all, but we ignore him*.

Against this background a question arises as to how to break the vicious circles of aggressiveness that some violence-stricken children seem to be entrapped in, and, in particular in our study, to which extent play can contribute to this aim. Interestingly, despite the fact that children unanimously identified play as a source of personal and social wellbeing and an important factor in striving for inner and social peace (contributing also, at least in some cases, to peaceful conflict resolution), when asked whether “playing together could help children who are always fighting to make peace” opinions were split. Children engaged in lively discussion, and when asked to vote, roughly half of them said *yes: play can help to make peace* while the other half said *no, it cannot*. This is how they justified their opinions:

*B: I think yes, because they [children who fight] can play with more children there, and end up being friends and all that—by playing*.

*G: I think no because we have classmates who, when we play football and they lose or something like that, or for example they win, they make fun of us and then if they lose, they get angry and start crying*.

Some children were of two minds about the matter:

*G: I think yes, but no, because sometimes R. and I fight, but sometimes we stop being friends, but we don't stop being friends, we talk again later*.

Some children suggest that those children *who always fight* should play only certain types of games (for example, those less competitive or games that do not involve close physical contact) as a way of avoiding conflicting situations:

*G: I recommend playing tag because I feel like it's something you can't get angry about because we're all playing, and if you push us, it will obviously be unintentional because it's tag*.

However, other children did not agree, locating the source of conflict outside the circumstantial conditions of play:

*G: They can play hide and seek, they can play tag, they can play lots of games, but that doesn't mean they're not going to fight*.

Perhaps, the best summary of children's discussion is the following opinion of one girl:

*G: I say no first of all because (…) in my course we all hit each other. Once two people hit each other, they're going to hit each other forever. Forever, unless you take them to a psychologist. Except for my friends and me, we sort it out when we leave*.

This controversy seems to confirm that children are both very resilient and, at the same time, sensitive to violence-related adversities. Many of them displayed a healthy attitude to the omnipresent violence and conflict by being able to sort out their conflicts in a peaceful manner: through conversation *(I feel that there are quite a few children who fight, who can resolve things by talking them over)*, through play (*because [through play] they would get to know each other better and wouldn't have conflicts*), or by letting bygones be bygones (*To resolve it [a conflict], we keep playing, we leave it at that*). Some adopted non-violent attitudes, remaining passive witnesses of ongoing fights, *ignoring* classmates who *always insulted*, or distancing themselves when conflicts occurred (*I left because I didn't want any trouble*). At the same time, they were never free from fear of political violence (they reported feeling *anguished because you never know when the same thing that happened to other people will happen to you*) and reported experiencing peer violence at school on a *daily basis*. This might be the reason why, despite valuing peace and harmony and actively seeking it, these violence-stricken children ended up entrapped in vicious circles of aggression that crept into their mindsets (as one girl said, for her hitting is a form of showing affection to her friends: it *is like our love*, as she put it). Therefore, as was obvious to the children themselves, in some serious cases, the transition to a non-violent life cannot be made without adults' support and professional psychological help.

## Discussion

The story of children migrants and refugees coming from conflict zones is a dual one. On the one hand it is a story of trauma, tragedy and heart-wrenching suffering. In conflict zones, children not only live under constant threat and witness violent events, but are increasingly becoming direct victims of political violence. Contemporary wars, unlike those of the past, fought by regular soldiers on the battlefield, are creeping into the very heart of civil communities, deliberately targeting the most vulnerable by attacking orphanages (Kiev) or schools (Gaza Strip). In many Latin American countries, children are experiencing similar traumas even in the absence of military conflict in the strict sense. As UNICEF (Gregson, 2024, p. 3ff; Cf. UNICEF, 2025) reports, the region of Latin America and Caribbean (LAC) has the highest child and adolescent homicide rate globally due to armed violence (such as criminal and interpersonal violence, internal disturbances or tensions, riots, acts of banditry). In this part of the world, a child is killed every 70 min (UNICEF and Plan Eval, 2023, p. 4). In our study children reported being constantly exposed to various mutually reinforcing forms of political and social violence, which also deliberately target children (by, for example, child abductions and political detentions under the Maduro regime), by invading their residential settlements, making them witness brutal events (Venezuela as *pure gunfire*) and, similarly to the child victims of war, exposing them to the hardship of forced displacement.

Although in terms of negative peace, Latin America is doing better than countries affected by direct armed conflicts, in terms of positive peace, the region is lagging behind (Global Peace Index, 2024, p. 8). Perhaps the biggest problem in this part of the world is the widespread, endemic armed violence committed by criminal organizations (many of which are often politically motivated [cf. Messari, 2024]), as well as extreme poverty and social injustice (Gregson, 2024). As (Galtung 1969) made clear, positive peace cannot be attained without counteracting structural violence in form of “unequal life chances” (p. 170–171) and cultural violence any aspect of a culture that can be used to legitimize violence (Galtung, 1990, p. 291). As demonstrated in our study, children defined peace both in negative terms (lack of armed conflict of *necessity to use weapons*, and lack of *fights, at school, in the streets or, I don't know, anywhere*) or as a positive striving toward social justice, solidarity, mutual aid and healthy social environment. Their deep understanding of positive peace and its various components may be related to the fact that children experienced deep-rooted poverty and structural violence on their own skins and correctly identify it as a factor that often leads to the use of weapons and other forms of social and political unrest. As for cultural violence, Latin America is fueled by postcolonial cultural norms that legitimize the class division, or, as in Chile, feed the growing anti-immigration sentiments (Pavez-Soto, 2018). The lack of positive peace on political and social levels also affects interpersonal violence in homes and communities (e.g., child and female abuse, rape, robbery, assault, homicide), which were also reported by the children in our study.

There is no doubt that the brutal ways by which political and social conflict affects its youngest victims leave lasting traces on their psychological and social wellbeing. In the aftermath of traumatizing, violence-related events, child migrants and refugees suffer from posttraumatic stress disorder, depression and anxiety disorders, sleep problems, chronic stress, aggressive behaviors, school refusal, loss of appetite and other somatic symptoms (Slone and Mann, 2016; Blackmore et al., 2020; Amiri, 2022). The gravity of these symptoms varies from being discomforting, to disturbing or seriously disabling a child's functioning (UNICEF, 1993). In our study children reported living under constant stress, and experiencing many troubling emotions, such as *sadness, solitude, anxiety, grief* , *rage, frustration, feeling depressed*, and *in pain*. Perhaps the most troubling and potentially dangerous outcome of violence exposure in this case is the children's increased aggressiveness. There is consistent evidence that exposure to both political violence (cf. Docherty et al., 2023; Huesmann et al., 2016; Landau et al., 2025; Dubow et al., 2009; Kithakye et al., 2010), as well as social or interpersonal violence (cf. Wahl and Metzner, 2011; Guerra et al., 2003; Schwartz and Proctor, 2000) leads to later habituated aggressiveness among children, which was evident in our study. The mechanisms of influence of political and social violence on children's aggressiveness were not the focus of our research, but it quickly became clear from their reports that many children, both in the group and in the children's environment, had been caught in the vicious cycles of aggression. Children's internalized violence and aggressiveness makes them potentially more vulnerable to recruitment by criminal organizations, which, sadly, is widespread in the LAC region, especially in case of children living in challenging socio-economic conditions (UNICEF and Plan Eval, 2023, p. 5).

However, the story of trauma, tragedy and unbearable suffering is only part of child migrants' story. As (White 2006) puts it, “there is also a second story of how the child has responded to these experiences of trauma, and this second story is often overlooked. No one is a passive recipient of trauma. Even children respond in ways to lessen the effects of the trauma” (p. 87). Although it may seem that the tragic events of armed conflict and armed violence, especially when experienced at a young age, are impossible to overcome, children often demonstrate an astounding resilience in the face of the utmost adversity (Fernando and Ferrari, 2013). Thus, the second story is a tale of children's undying hope, joy, and their capability to actively transform scenarios of tragedy into testimonies of personal resilience and agency. Focusing solely on deficits or psychopathology threatens to entrap young victims in the overwhelmingly tragic narrative and, as (Veronese and Barola 2018, p. 315; cf. Bracken, 2001) put it, “a sort of monological sense of self, which is exclusively informed by trauma, loss, and dispossession.” The call for a second story is thus a call to break the deterministic power of the trauma narrative over children's lives. It is important to mention in this context that the resilience perspective is not an alternative nor antidote to the horrors of armed conflict and violence, but rather a way to complete and counterbalance trauma-focused interventions with practices that build on children's natural survival and coping skills. Both storylines are irreducibly interwoven into the lives of child migrants and refugees, requiring adults' increased efforts to protect, as well as support their internal resources to rise above tragic scenarios.

Our study confirms the children's resilience and their agency in transforming tragic scenarios into scripts of hope and joy. The children in our study, despite having passed through many difficult experiences, retained their childlike innocence, trust and openness. They clearly valued friendship, were capable of engaging in meaningful relationships, trusted newcomers and welcomed them in their groups. They also showed a lot of empathy toward others' suffering (especially the most harmless ones such as animals) and eagerly helped one another in need. Remarkably, despite having experienced different forms of violence on a daily basis (and sometimes engaging in aggressive behaviors themselves), they adopted a very profound understanding of peace and actively sought to create a culture of peace in their communities, and were able to adapt non-violent attitudes and solve their own conflicts through conversation or play. Even if we often think of resilience as an individual trait, it is important to highlight that it will not develop without active support from children's social environment (National Scientific Council on the Developing Child, 2015, p. 1 ff). Children in our study were embedded in a supportive social context: all child migrants had homes with at least one parent present in Chile, they attended school and reported enjoying it, and, in general, belonged to groups of friends in which they actively supported each other.

An indispensable part of children's resilience is their agency. According to the definition of (Cavazzoni et al. 2022, p. 1126), agency is the capacity to act and mobilize resources to preserve one's own wellbeing. This falls short of (Panter-Brick and Leckman 2013) definition of resilience as a process of harnessing biological, psychosocial, structural, and cultural resources to sustain one's own wellbeing. The children in our study actively sought means to maintain their own wellbeing, among which play, friendship and spending time with beloved creatures (including animals) were the main mechanisms. Their agency was also evident in the way they valued their participation rights and empowerment gained through learning that they are subjects of rights. Recognizing children as agents capable of creating meaning, overcoming hardships, and mobilizing resources that serve their wellbeing, as well as being engaged actors in their social environments, directly corresponds to the conception of childhood underlying the UN CRC, which emphasizes children's participatory rights next to these which guarantee their protection and provides them with the best possible services.

The main claim of this paper is that one of children's most important resilience-enhancing strategies is play. Including play in the resilience framework for young victims of war and violence has the advantage that, as children's natural way of being and one of their most favorite activities, it is probably the most attractive and cost-effective resilience-oriented intervention. The very heart of the attraction of play is the ecstatic feeling of liberty (Gray, 2013, p. 141) to design their own alternative scenarios, roles and destinies in the adult-ruled world. Emphasizing the agentic nature of play, (Lester and Russell 2010, p. 15ff) described it as a form of “self-protection” or “self-resilience,” whereby children can be authors of their own wellbeing and protagonists of their own stories. The children in our study reported abundantly on the many ways in which they actively and consciously deploy play to serve their own wellbeing, for example to *relieve stress, forget about problems, distract the mind*, and to *recall good memories*. This nature of play, by definition both agentic and participatory, corresponds to the concept of play found in the children's rights framework, in which as the UN Committee on Children's Rights put it, it can serve a “natural, self-guided and self-healing process” (CRC, 2013, p. 39) of trauma recovery and social reintegration. Remarkably, child migrants and refugees in our study saw the benefits of play in their lives very similarly to the UN Committee, that is, as contributing to their physical and psychological recovery and social reintegration. With surprising maturity, the children emphasized the importance of play for their holistic health, mental recovery and the development of social relationships. Play served as an instrument for reintegration into a new country thanks to its inclusive, friend-making and culture-bridging potential. Moreover, the children emphasized that the right to play was an important factor contributing to their inner and social peace.

Despite this multifaceted potential of play to allow children to flourish, our study also pointed out some of its limitations. The dynamics of play can give rise to many situations that cause physical or psychological distress: children run, bump into each other and collide; they can also win, lose, cheat, play unfairly or experience injustice. As such, it can activate the habitual aggressiveness of children affected by violence. This shows that although play can indeed serve as a “natural” and “self-guided” means of children's post-conflict recovery and social reintegration, it cannot be deployed indiscriminately in resilience programs. Interestingly, the children in our study astutely proposed several possible interventions, such as engaging children *who always fight* in more peaceful and cooperative games instead of competitive and aggressive ones, and, in more serious cases, offering troubled, aggressive children psychological help. Since many children in our study reported problems with solving their disputes, it would be very helpful to provide children in conflict zones or in post-conflict situations with programs that strengthen their capacities for non-violent conflict resolution (Clayton et al., 2001; Johnson and Johnson, 2006; Guetta, 2020).

## Conclusion

This study confirmed that, just as identified by the Committee on the Rights of the Child (CRC, 2013, p. 39), play can contribute to the psychological recovery and reintegration of child victims of political and social violence. Moreover, play in our study turned out to be a powerful means through which children shared their stories, experiences, and often remarkably profound opinions with us. Children very generously invited us into their worlds, relating political and social struggles in their countries of origin, recalling difficulties of the perilous journey to safety and sharing dreams for a better future in Chile. As said, the story of children migrants and refugees in our study is a dual narrative: it is, on the one hand, a story of violence and trauma that nobody, especially the youngest members of our human community, should be exposed to, and, on the other hand, a story of hope, resilience, friendship and joy. Despite the many hardships, challenges and internal struggles they face, the children in our study demonstrated deep compassion toward the suffering of others, especially those more vulnerable than them: animals, bereaved children and children of war, whose voices some of them chose to represent in the Children's Congress. As carriers of children's vulnerable and unrecognized voices we would like to honor their experience and wisdom by formulating some research-related and practical recommendations with which we think children in our study would agree, and which, we hope, may benefit child migrants and refugees around the world.

Let us start with some recommendations for future research. There is a need to further explore the healing potential of play for children affected by violence. Although the therapeutic potential of professional play therapy is well evidenced in literature (cf. Cohen and Gadassi, 2018; Cohen, 2014, 2019; Cohen et al., 2010; Bratton et al., 2005; Lin and Bratton, 2015; Ray et al., 2015), research on the therapeutic effects and potential of play in children's natural environments, such as school, home, neighborhood is less systematic. Considering that the socio-economic situation of migrants and refugees is often precarious (which can result in a lack of access to professional psychological play therapy), there is a need to examine more thoroughly the conditions needing to be met for everyday play to fully develop its therapeutic role for traumatized children.

Moreover, our exploration of the potential of play as an instrument of peace education and peace-building is a novel contribution to the field, which we hope will be explored in future studies. There is a long tradition of treating sport as a potential instrument of social peace –as is recognized in the UN-led programs on Sport for Development and Peace (cf. Burrmann et al., 2017; Adler et al., 2018; Doidge et al., 2020; Flensner et al., 2020; Zimmermann and Morgan, 2020). However, the role of non-formal sport and other forms of play is still under-recognized (cf. Gray, 2013; Sterchele, 2015; Koopmans and Doidge, 2021). The children in our study demonstrated profound understanding of different dimensions of peace, including the internal one, which is still barely present in peace studies, in particular in relation to children (cf. Balvin and Christie, 2020; Guetta, 2020). Our study revealed the empowering potential of children's rights, especially of their right to play (and playful participation) and their rights to be heard. Children not only want and need to be listened to, but also, as a girl in our study put it, they are *the ones with good ideas*. We hope that these findings will contribute to seeing children as agents of peace in their communities and their increasing inclusion in peacebuilding, recovery and reconciliation programs in violence-stricken communities.

Regarding practical recommendations, we would like to advocate for the greater and more systematic provision of children's right to play in vulnerable settings, and its inclusion to peace-building programs, both on international and national levels. Consistently with the UNICEF and Plan Eval (2023) recognition of the importance of children's rights for peace, there is a need for further systematic programming on children's right to play as a peace-enhancing strategy in conflict zones. Such an international policy should include minimum standards for providing opportunities for play, as well as guidelines for training staff and volunteers that sensitize them to the problems that can arise during post-traumatic play of children affected by violence (such as their habituated aggressiveness, post-traumatic stress disorder, and other mental problems). Also, despite the best effort of the non-governmental organizations, their initiatives remain scarce, insufficiently researched in terms of optimal play strategies and the evaluation of their outcomes, underfunded and, above all, still seen as optional –an ancillary provision compared to other more urgent children's needs (Ager et al., 2013; Metzler et al., 2015; Collins and Wright, 2019; Ardelean, 2021; Heldal, 2021). There is also a need for a consolidated public policy of countries receiving migrants and refugees both in Latin American and Caribbean countries and in other regions of the world to fulfill their obligations under Article 31 of the UNCRC toward children seeking shelter in their territory. The therapeutic, empowering and peace-building potential of this right, as practiced and demonstrated in this study, shows that engaging the youngest in peace-building activities through play is the beginning of a better future for all of us. We also encourage public schools, as well as other educational and non-educational institutions serving migrant and refugee children, to prioritize play as a tool for integration, for mental health support, and for coping with post-traumatic stress. However, these institutions must also receive proper training and adopt trauma-informed perspectives to create safe, inclusive environments that prevent discrimination and retraumatization. Ultimately, all adult stakeholders—including policy makers, politicians, educators, and other individuals—share the critical responsibility of breaking cycles of violence and fostering a future where every child can thrive.

## Data Availability

The data sets obtained during this study are not publicly available due to ethical restrictions. They will be made available upon individual request for legitimate reasons. Requests for access should be directed to AG [aleksandra.glos@uc.cl].
